# TCHL – a phase II neo-adjuvant study assessing TCH (docetaxel, carboplatin and trastuzumab) and TCHL (docetaxel, carboplatin, rastuzumab and lapatinib) in HER-2 positive breast cancer patients: a 5-year follow-up with serum biomarker analysis

**DOI:** 10.2340/1651-226X.2025.43143

**Published:** 2025-06-05

**Authors:** John Crown, Alex J. Eustace, Denis M. Collins, Maccon Keane, Linda Coate, John Kennedy, Seamus O’Reilly, Catherine Kelly, Miriam O’Connor, Michael Martin, Conleth Murphy, Karen Duffy, Janice Walshe, Giuseppe Gullo, Thamir Mahgoub, Alberto Alvarez-Iglesias, Imelda Parker, Vicky Donachie, Ausra Teiserskiene, Stephen F. Madden, Brian Moulton, Norma O’Donovan, Bryan T. Hennessy

**Affiliations:** a Department of Medical Oncology, St Vincent’s University Hospital, Dublin, Ireland; b Life Sciences Institute, Dublin City University, Dublin, Ireland; c School of Biotechnology, Dublin City University, Dublin, Ireland; d Cancer Biotherapeutics Research Group, School of Biotechnology, Dublin City University, Dublin, Ireland; e Department of Medical Oncology, Galway University Hospital, Galway, Ireland; f Department of Medical Oncology, University Hospital Limerick, Limerick, Ireland; g Department of Medical Oncology, St James’s Hospital, Dublin, Ireland; h Department of Medical Oncology, Cork University Hospital, Cork, Ireland; i Department of Medical Oncology, Mater Hospital, Dublin, Ireland; j Department of Medical Oncology, Waterford Regional Hospital, Waterford, Ireland; k Department of Medical Oncology, Sligo University Hospital, Sligo, Ireland; l Department of Medical Oncology, Bons Secours Hospital, Cork, Ireland; m Department of Medical Oncology, Letterkenny University Hospital, Donegal, Ireland; n HRB-Clinical Research Facility, Galway, Ireland; o Cancer Trials Ireland/ICORG, Dublin, Ireland; p Data Science Centre, Royal College of Surgeons in Ireland, Dublin, Ireland; q Clinical Oncology Development Europe, Dublin, Ireland; r Department of Medical Oncology/ICORG/Cancer Trials Ireland, Beaumont Hospital, Royal College of Surgeons in Ireland, Dublin, Ireland

**Keywords:** HER2-positve breast cancer, neoadjuvant treatment, trastuzumab, lapatinib, tyrosine kinase inhibitors, cancer biology

## Abstract

**Background:**

The docetaxel (T), carboplatin (C) and trastuzumab (H) regimen has been used in the (neo-) adjuvant treatment of HER2+ early stage breast cancer (ESBC). Lapatinib (L) a small molecule HER2 antagonist produces clinical responses following H failure.

**Methods:**

We randomly assigned 88 patients with stages Ic–III HER2+ESBC to receive neoadjuvant TCH, TCL or TCHL followed by surgery and 1 year of H. The primary endpoint was pathological complete response (pCR). Secondary objectives were overall and disease-free survival (OS, DFS).

**Results:**

The TCL arm was closed following demonstration of inferiority of L in another trial. The pCR rates for TCH and TCHL were 52.8 and 51.6 (*p* = 1.0). At a median 4.8 years follow-up, TCHL patients had a significantly superior DFS; however, OS was similar. Prophylactic loperamide reduced the frequency of diarrhoea. Serum biomarker analysis identified a link between high tumour T-cell levels and high red blood cell, haematocrit, and haemoglobin following commencement of therapy.

**Interpretation:**

The study did not meet its primary endpoint of superior pCR. TCHL produced a significant improvement in DFS. Our study and others suggest a possible role for L in neoadjuvant therapy of HER2+ ESBC.

**Clinical Trial Registration:** NCT01485926.

## Background

Breast cancer is the most common potentially fatal cancer in women. Approximately 2,400 new cases of breast cancer are diagnosed annually in Ireland (www.ncri.ie), and 379,000 in the European Union (www.ecis.jrc.ec.europa.eu). Surgery and radiotherapy can often cure breast cancer limited to the breast and axillary nodes [1]. However, some patients later develop metastases due to undetected spread before surgery. Adjuvant systemic therapy aims to eliminate these micro-metastases [2]. Chemotherapy, endocrine therapy, targeted agents, and immune checkpoint inhibitors help reduce relapse and improve survival [1, 3, 4]. Systemic treatment is especially important in HER2-postive (HER2+) cancers, which have a higher risk of recurrence [5–7]. In these cases, adding trastuzumab, a monoclonal antibody targeting HER2, to adjuvant therapy has led to significant improvements in disease-free and overall survival (OS) [8].

Systemic treatment given pre-operatively (neo-adjuvant) can target micro-metastases early and may shrink tumours, enabling less extensive surgery [9]. This approach is particularly effective in HER2+ cancers, where many patients achieve complete pathological remission with chemotherapy and trastuzumab – an outcome linked to excellent prognosis [10]. The addition of a second anti HER2 monoclonal antibody, pertuzumab [11] and antibody–drug conjugates like T-DM1 and trastuzumab deruxtecan further improve outcomes in HER2+ breast cancer [12, 13].

While trastuzumab targets the extracellular domain of the HER2 protein (a transmembrane tyrosine kinase receptor), other ‘small molecule’ agents have been developed which target the intracellular component. The first of these to enter clinical trials was lapatinib, a molecule which also targets the epidermal growth factor receptor. In a large phase III trial conducted in patients with HER2 altered metastatic disease, whose cancer had progressed despite trastuzumab-containing therapy, the combination of the chemotherapy drug capecitabine plus lapatinib was superior to capecitabine alone [14]. This finding is likely explained by the fact that trastuzumab and lapatinib are susceptible to different resistance mechanisms. There is synergy between these agents *in vitro*, and this synergy is supported by the observation that in patients whose disease has become resistant to trastuzumab, the combination of trastuzumab and lapatinib was superior to lapatinib alone [14–18].

We conducted a prospective random assignment phase II trial to compare the relative efficacy of trastuzumab (H), lapatinib (L) or the combination of these agents together with carboplatin (C) and docetaxel (T) chemotherapy as neo-adjuvant therapy for patients with early-stage breast cancer. We also performed translational studies of potential predictors of response.

### Participants

Eligible patients had biopsy-proven HER2+ breast cancer. HER2 positivity was defined by 3+ immunohistochemistry, or, HER2 fluorescent in-situ hybridization with an amplification ratio >2.0. Patients with distant metastases were excluded. Patients had to have adequate organ function, an ECOG performance status of 0–1, and no active heart disease. Patients must not have had prior definitive breast surgery, definitive breast surgery following neoadjuvant treatment. Patients must have had a tumour, node metastasis classification system (TNM) stage (refer to AJCC 7th Edition – Appendix M) of breast cancer which included T2, T3, T4a, T4b, T4c, or T4d and node negative or node positive (histologically or cytologically confirmed) or, any tumour with lymph node positive disease (histologically or cytologically confirmed). Patients with multifocal tumours are not excluded; tumour stage assignment must have been based on the largest tumour. Patients presenting with bilateral breast cancer are not eligible. Full eligibility criteria and the clinical trial protocol are available at NCT01485926 (www.clinicaltrials.gov).

### Interventions

**Figure 1:**
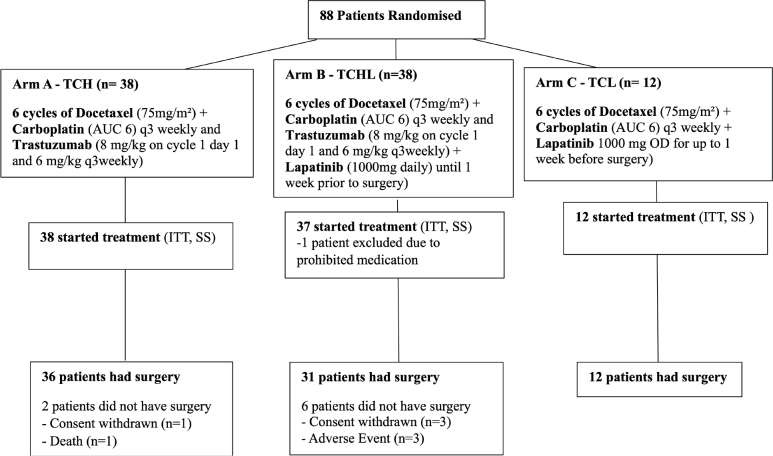
Participant flow chart: populations added. ITT: Intent to Treat; SS: Safety Set.

Patients were treated with six cycles of C and T chemotherapy together with anti-HER2 therapy. Patients in Arms A, B, and C received H alone, H and L, and L alone as pre-operative anti-HER 2 therapy respectively (Figure 1). When the results of the NCIC CTG MA.31 [19], indicating inferiority for a L alone arm, became available, we closed our Arm C (L alone). Patients who were previously randomised to Arm C and who were still on neo-adjuvant treatment with TCL had H added to their regimen. Patients who had completed neo-adjuvant treatment with TCL and were on H maintenance therapy followed the protocol schedule, received H for 1 year from first dose of H.

Patients then had definitive cancer surgery, either breast conservation or mastectomy and axillary dissection or, sentinel node surgery. All patients had postoperative adjuvant H to complete 1 year of H treatment. Patients with hormone receptor-positive disease received post-operative adjuvant endocrine therapy according to institutional criteria. Radiotherapy was given per institutional criteria.

The study had its first patient enrolled on the 24th December 2010, with the last patient being enrolled on the 08th May 2013. The last patient came off study after 5 years on 22nd June 2018 as required per protocol. Median follow-up on the study was 4.8 years (58.8 months), ranging from 1 day (patient withdrew due to study non-compliance) to 6.8 years.

Use of prophylactic growth factors was a mandatory requirement for all patients on the study, and all patients must have received granulocyte colony-stimulating factor (GCSF) as a primary prophylaxis for febrile neutropenia whilst receiving chemotherapy.

Peg-filgrastin (Neulasta) was recommended; however, filgrastin (Neupogen) may have been used as a substitute for patients who suffered extreme bone pain. The dosing for Neulasta was 6 mg subcutaneously for 24 hours following each cycle of chemotherapy (not to be given greater than 72 hours after chemotherapy dose). Neupogen dosing was 5 μg/kg subcutaneously for 7–10 days beginning on day 2 after chemotherapy.

Loperamide was given prophylactically to reduce the number and severity of diarrhoea-related adverse events expected with the treatment of TCL and TCHL. Patients were treated from day 1 onwards with a dose of 4 mg/day of loperamide, followed by 2 mg after each loose motion, up to a maximum of 10 mg in 24 hours.

### Outcomes

The primary objective was to assess the efficacy of TCH, TCL, and TCHL in the neo-adjuvant treatment of HER2+ breast cancer, using pathological complete response (pCR) as the primary endpoint. pCR is defined by the absence of invasive carcinoma in the breast and lymph nodes, as per AJCC 7th Edition. Secondary endpoints were disease-free survival (DFS) and OS.

In total, eight patients discontinued the study before surgery due to: death (*n* = 1 TCH patient), withdrawal of consent (*n* = 4, 1*TCH, 3*TCHL patients), and an adverse event (*n* = 3 TCH patients). The above eight patients are therefore not evaluable for pCR.

### Sample size

A sample size of 36 evaluable patients was required to detect an absolute 25% difference in the pCR rate between the hypothesised 65% pCR rate versus the historical-control pCR rate of 40%, with a nominal one-sided 0.05 significance and 91% power using the exact binomial method. Assuming a 5%–10% non-evaluability rate typical for such pilot studies, 40 patients were planned to be enrolled to each treatment arm, thus 120 patients in total were to be enrolled into the Phase II study.

### Statistical methods

All the statistical analyses were carried out in R statistical software (version 3.5.1).

All eligible patients were included in the Intention to Treat (ITT) analysis. All patients proceeding to surgery following neoadjuvant treatment were included in the efficacy analysis. All patients who had at least one cycle of therapy were included in the safety analysis.

pCR rate is defined as the proportion of patients who achieve pCR at the time of definitive surgery (after 18 weeks).

OS is defined as the time from the date of registration until death from any cause. Patients who are withdrawn before the time of the analysis were censored at the time they were last known to be alive.

DFS is defined as the time from the date of registration until the date of relapse, second primary malignancy, progression (including death). If the subject did not progress or die at the time of the analysis, the DFS time was censored at the day of their last tumour assessment.

The primary endpoint of pCR was analysed using the ITT set using a one-sided binomial test. A further sensitivity analysis of pCR was performed by randomisation strata (tumour size (≤3 and >3 cm) and by hormone receptor status (estrogen receptor [ER] and/or progesterone receptor [PR] positive versus ER and PR negative) using Fisher’s Exact test.

A secondary objective (secondary endpoint) for the TCHL study was to assess the DFS and OS between TCH, TCL, and TCHL in HER-2 positive breast cancer using the ITT Set. The log-rank test was used to compare the survival times of the treatment arms. Further translational objectives were to assess the relationship between drug exposure and adverse events using the Safety Set and to examine any potential molecular and pharmacological markers of response to H and L.

We also aimed to determine if prophylactic loperamide significantly reduced the number of diarrhoea–related adverse events. For loperamide, the comparison is between the groups of ITT TCHL/TCL patients who started treatment when use of loperamide was not mandatory versus the ITT TCHL/TCL patients who started treatment when use of loperamide was mandated. To compare these two pre- and post-mandate groups we used the Fisher’s Exact Test and negative binomial regression models for rates.

The translational objectives in the study were to examine associations between tumour infiltrating lymphocytes (TILs) and blood count markers associated with peripheral oxygen carrying capacity.

### Biomarkers

CD3+, CD4+, and CD8+ immune cell subsets were determined in pre-treatment and on-treatment (20 day) formalin-fixed paraffin-embedded (FFPE) tumour blocks from *n* = 13 patients. A detailed description of the methods employed is available in the associated translational paper [20]. Briefly, 4 μm serial FFPE sections were stained for CD3 (Leica, NCL-L-CD3-565), CD4 (Leica, NCL-CD4-368), and CD8 (Leica, NCL-CD8-4B11). Detection and visualization of stained cells was achieved using the Bond Polymer Refine Detection Kit (Leica, DS9800) with Bond DAB Enhancer (Leica, AR9432). Tissues were counterstained with haematoxylin and cover slipped. Slides were scanned at 40× using a Philips 2.0 scanner and the whole section was analysed using the open access image analysis software QuPath [21]. The positive cell detection tool was used to measure the number of positive cells per square millimetre of tissue and compared against the assessment of a histopathologist to ensure software accuracy in advance of using the QuPath results in analyses.

### Blood counts

Standard bloods were taken in advance of each cycle of therapy as per the clinical trial protocol. The red blood cell count (RBC) (million cells/mcL)), haemoglobin (g/dL), and haematocrit (% of blood) values are utilised in this study.

## Results

### Baseline patient characteristics

Eighty-eight patients were registered to the TCHL clinical trial and their baseline characteristics are summarised in Table 1. One patient with a major protocol violation was deemed ineligible and was not treated on study. The remaining 87 patients (Arm A = 38, Arm B = 37, Arm C = 12) were registered, randomised, and included in the ITT analysis. A total of 79 patients (Arm A = 36, Arm B = 31, Arm C = 12) were evaluable for the surgery related efficacy analyses. The 87 patients in ITT are assessed for survival efficacy endpoints, not just response. Eight patients, two in Arm A (consent withdrawn = 1, death = 1) and six in Arm B (consent withdrawn = 3; adverse event = 3) did not have surgery and were not evaluable for response. All 87 patients were evaluable for Safety (Figure 1).

**Table 1:** Baseline clinico-pathological characteristics treated in the TCHL clinical trial.

Clinico-pathological characteristic		Total – 88	Treatment arm		
			TCHL – 38	TCH – 38	TCL – 12
		*n* (%)	*n* (%)	*n* (%)	*n* (%)
Age	Mean ± SD	50.5 ± 9.6	50.3 ± 11	50.7 ± 8.7	50.1 ± 8.2
ECOG status	0	76 (86.4%)	33 (86.8%)	32 (84.2%)	11 (91.7%)
	1	12 (13.6%)	5 (13.2%)	6 (15.8%)	1 (8.3%)
ER/PR status	ER and PR negative	32 (36.4%)	14 (36.8%)	15 (39.5%)	3 (25%)
	ER and/or PR positive	56 (63.6%)	24 (63.2%)	23 (60.5%)	9 (75%)
Breast cancer type	Invasive ductal carcinoma	81 (91.1%)	35 (91.1%)	34 (90.0%)	12 (100%)
	Other	7 (7.9%)	3 (7.9%)	4 (10.0%)	0 (0%)
T status	T1	9 (10.2%)	3 (7.9%)	5 (13.1%)	1 (8.3%)
	T2	51 (57.9%)	22 (57.9%)	21 (55.4%)	8 (66.7%)
	T3	16 (18.3%)	9 (23.7%)	7 (18.4%)	0 (0%)
	T4	12 (13.6%)	4 (10.5%)	5 (13.1%)	3 (25.0%)
N status	NX	4 (4.5%)	2 (5.3%)	2 (5.3%)	0 (0%)
	N0	25 (28.4%)	10 (26.3%)	11 (28.9%)	4 (33.3%)
	N1	55 (62.5%)	22 (57.9%)	25 (65.8%)	8 (66.7%)
	N2	2 (2.3%)	2 (5.3%)	0 (0%)	0 (0%)
	N3	2 (2.3%)	2 (5.3%)	0 (0%)	0 (0%)
M status	M0	79 (89.8%)	35 (92.1%)	33 (86.8%)	11 (91.7%)
	MX	9 (10.2%)	3 (7.9%)	5 (13.2%)	1 (8.3%)
Stage	I	1 (1.1%)	0 (0%)	1 (2.6%)	0 (0%)
	II	64 (72.8%)	29 (76.3%)	26 (68.4%)	9 (75%)
	III	22 (25.0%)	9 (23.7%)	10 (26.4%)	3 (25%)
	Unknown	1 (1.1%)	0 (0%)	1 (2.6%)	0 (0%)

The arms of the study were well-balanced in regard to patients age, ECOG performance status, TNM status. Hormonal receptor status was recorded for each tumour with 63.6% (*n* = 56) having expression of either hormone receptor (HR) status or PR. A total of 36.4% (*n* = 32) of tumours had neither expression of ER or PR. The treatment arms were similar in respect to hormone receptor expression. The trial recruited patients who mainly presented with either Stage II (72.8%; *n* = 64) or Stage III disease (25%; *n* = 22), and no significant differences were observed between the frequencies across the treatment arms.

### Clinical outcome

At the time of suspension of Arm C, 12 patients had been randomised and 8 patients had completed their TCL treatment. The remaining four patients had H added to their neoadjuvant treatment. All Arm C patients completed 1 year of adjuvant H.

The TCHL study failed to meet its primary endpoint, as there was no statistical difference in pCR rates between the TCH, TCL and TCHL arms (*p* = 0.2). The overall pCR rate was 49.4% with a further 39.2% of patients achieving a partial response (PR) and only 11.4% of patients having no response (NR) to their treatment (Table 2). When we assessed the pCR rates between the treatment arms that remained active throughout the study (TCHL and TCH) the pCR rate for TCHL group was 51.6% whilst it was 52.8% for the TCH regimen (Diff = –1.2%; 95% CI: 25%–22.8%; *p*-value: 1.000).

**Table 2:** Analysis of rates of pathological compete response (pCR) in the TCHL clinical trial for the per protocol set.

pCR response	Overall, *N* = 79^a^	Treatment Arm			*P* ^b^	TCHL TCH p*
		TCHL, *N* = 31^a^	TCH, *N* = 36^a^	TCL, *N* = 12^a^		
**Response**					0.2	1.000
pCR	49.4% (39/79)	51.6% (16/31)	52.8% (19/36)	33.3% (4/12)		
pPR	39.2% (31/79)	45.2% (14/31)	33.3% (12/36)	41.7% (5/12)		
pNR	11.4% (9/79)	3.2% (1/31)	13.9% (5/36)	25.0% (3/12)		
**ER and PR** –					0.7	0.429
pCR	65.5% (19/29)	75.0% (9/12)	57.1% (8/14)	66.7% (2/3)		
non-pCR	34.5% (10/29)	25.0% (3/12)	42.9% (6/14)	33.3% (1/3)		
**ER and/or PR +**					0.4	0.531
pCR	40.0% (20/50)	36.8% (7/19)	50.0% (11/22)	22.2% (2/9)		
non-pCR	60.0% (30/50)	63.2% (12/19)	50.0% (11/22)	77.8% (7/9)		

ER: Estrogen receptor; PR: Progesterone receptor; pPR: Pathological Partial Response; pNR: Pathological No Response.

a % (n/N).

b Fisher’s exact test.

* *P* value from TCHL TCH analysis.

Across the study, patients who had tumours that were hormone receptor negative (ER/PR negative) had a pCR rate of 65.5%, compared to 40.0% in those whose tumours were HR positive (Diff = 21.5%; 95% CI: 2.3%–45.3%; *p* = 0.132). For patients with HR negative tumours, the pCR rates were 75% and 57.1% respectively for TCHL and TCH (Diff = 17.9%; 95% CI: 17.8%–53.5%; *p* = 0.429). For patients whose tumours were HR positive the pCR rates were 36.8% and 50% respectively for TCHL and TCH (Diff = –13.2%; 95% CI: 43.3%–17%; *p* = 0.531).

The pCR rate in the HR negative population treated with the TCHL arm was 75% compared to 36.8% in those who were HR positive (Diff = 38.2% 95% CI 5.4%–70.9%; *p* = 0.066).

**Figure 2:**
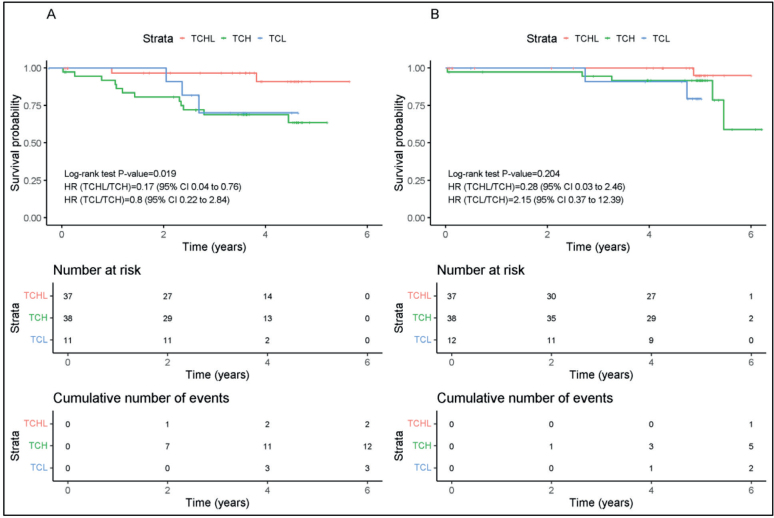
Kaplan-Meier survival analysis for (A) Disease-free survival (DFS) and (B) Overall survival (OS) for TCH (*n* = 38) (Green Line), TCHL (*n* = 37) (Red Line) and TCL (*n* = 12) (Blue Line) arms of TCHL study.

There was a difference in DFS between the TCHL, TCL, and TCH arms. Interestingly TCHL was associated with a significantly longer DFS than that observed in TCH treated patients (Hazard ratio (HR) 0.17, 95% CI: 0.04–0.76; *p* = 0.019). The median DFS had not been reached at the time of analysis since only 2 (5%) and 12 (32%) in the TCHL and TCH arms had progressed (Figure 2A).

The increased DFS did not correlate with a longer OS benefit in patients treated with either TCHL, TCL, or TCH. In our study OS was comparable between the TCH and TCHL groups (HR 0.28, 95% CI: 0.03–2.46); *p* = 0.204). Again the median OS was not estimable in our study (Figure 2B).

### Adverse events

In the TCHL study one patient who received TCH and who did not have protocol-mandated prophylactic granulocyte-colony stimulating factor died from neutropenic sepsis.

There was a non-significant increase in the incidence of serious AEs (SAEs) in patients who received L as part of their therapy (*p* = 0.069) (Table 3). The most frequent SAE was diarrhoea which occurred in 19.5% (*n* = 17) patients. Grade 3 diarrhoea occurred in 13 of these 17 patients. When different treatment arms were considered there was a significantly higher frequency of diarrhoea in patients who received the TCHL regimen; 32.4% (*n* = 12/37) versus those that received the TCH regimen 10.5% (*n* = 4/38) (*p* = 0.038) (Table 4).

**Table 3:** Summary of the number of patients who experienced at least one serious adverse event in the TCHL Clinical Trial and in the individual treatment arms.

Treatment arm	Total subjects	Any SAE				*P*
		Yes		No		
		*n*	%	*n*	%
TCHL	37	22	59.5% (22/37)	15	40.5% (15/37)	0.069
TCH	38	13	34.2% (13/38)	25	65.8% (25/38)	
TCL	12	7	58.3% (7/12)	5	41.7% (5/12)	
TOTAL	87	42	48.3% (42/87)	45	51.7% (45/87)	

SAE: serious adverse events.

**Table 4:** Summary of most common serious adverse events from the TCHL clinical trial using the safety analysis set (*n* = 87).

SOC	PT	Treatment arm	Total subjects		Grade					*P*
			*n*	%	1	2	3	4	5	
Gastrointestinal disorders	Diarrhoea	TCHL	12	32.4% (12/37)	2	-	10	-	-	0.038 *
		TCH	4	10.5% (4/38)	-	1	3	-	-	
		TCL	1	8.3% (1/12)	1	-	-	-	-	
		**TOTAL**	**17**	**19.5% (17/87)**	**3**	**1**	**13**	**-**	**-**	
	Vomiting	TCHL	4	10.8% (4/37)	-	1	3	-	-	NT
		TCH	1	2.6% (1/38)	-	-	1	-	-	
		TCL	1	8.3% (1/12)	-	1	-	-	-	
		**TOTAL**	**6**	**6.9% (6/87)**	**-**	**2**	**4**	**-**	**-**	
	Abdominal pain	TCHL	2	5.4% (2/37)	-	1	1	-	-	NT
		TCH	2	5.3% (2/38)	-	1	1	-	-	
		**TOTAL**	**4**	**4.6% (4/87)**	**-**	**2**	**2**	**-**	**-**	
	Nausea	TCHL	3	8.1% (3/37)	-	-	3	-	-	NT
		TCH	1	2.6% (1/38)	-	-	1	-	-	
		**TOTAL**	**4**	**4.6% (4/87)**	**-**	**-**	**4**	**-**	**-**	
Infections	Urinary tract infection	TCHL	2	5.4% (2/37)	-	1	1	-	-	NT
		TCH	1	2.6% (1/38)	-	-	1	-	-	
		TCL	1	8.3% (1/12)	-	-	1	-	-	
		**TOTAL**	**4**	**4.6% (4/87)**	**-**	**1**	**3**	**-**	**-**	
Metabolism nutrition disorders	Dehydration	TCHL	5	13.5% (5/37)	-	-	5	-	-	NT
		TCL	1	8.3% (1/12)	-	-	1	-	-	
		**TOTAL**	**6**	**6.9% (6/87)**	**-**	**-**	**6**	**-**	**-**	
Blood disorders	Febrile neutropenia	TCHL	1	2.7% (1/37)	-	-	1	-	-	NT
		TCH	2	5.3% (2/38)	-	-	2	-	-	
		TCL	1	8.3% (1/12)	-	-	1	-	-	
		**TOTAL**	**4**	**4.6% (4/87)**	**-**	**-**	**4**	**-**	**-**	
	Neutropenia	TCHL	1	2.7% (1/37)	-	-	1	-	-	NT
		TCH	2	5.3% (2/38)	-	-	1	1	-	
		TCL	1	8.3% (1/12)	-	-	-	1	-	
		**TOTAL**	**4**	**4.6% (4/87)**	**-**	**-**	**2**	**2**	**-**	

NT: Not Tested due to insufficient patient numbers; SOC: System Order Class; PT: Preferred Term and Grade (1–5).

*P*-values are calculated for the most common AEs, *n* ≥ 4, using chi-squared test, where * indicates a *p*-value <0.05.

Treatment with either TCHL, TCH, or TCL did not have a significant impact on patients cardiac function. The TCHL and TCH groups display a similar pattern in LVEF capacity over time with a regression slope near to 0 (Supplementary Figure 1A). LVEF function in the TCL arm appears to decrease over time; however, given the low number of patients in this group and the lack of values later in the study, those results cannot be used to conclude any patterns and should be treated with caution.

Regarding the number of cardiac events observed in the TCHL clinical trial, there are only nine recorded AEs for ‘Cardiac Disorders’ which account for a total of seven patients. One patient had a fatal event on the TCH arm, which was related to the study drug. No other patient withdrew consent for treatment due to cardiac toxicity or cardiac events (Supplementary Figure 1B, C).

The other most frequent SAEs recorded were vomiting and dehydration (both 6.9%), abdominal pain, urinary tract infection, neutropenia, febrile neutropenia, and nausea (4.6%) and hypokalaemia (3.4%) (Table 4).

### Assessment of prophylactic loperamide to reduce the incidence and severity of diarrhoea

Due to the high incidence of diarrhoeal events occurring in the TCHL trial it was mandated that loperamide should be included in the treatment schedule of patients to reduce the frequency and severity of these events.

When we assessed the number of patients with events in the TCHL arm, the introduction of mandated loperamide reduced the number of patients who had any grade of diarrhoea from 85.4% to 40.9% (*p* < 0.001).

We analysed the number of diarrhoea events that occurred during treatment before and after the introduction of the mandated loperamide for TCHL/TCL arms taking into account the varying length of treatment between patients.

There were 27 patients who had completed their treatment before the addition of the mandated loperamide, 14 patients were treated before and after the mandate, and 8 patients received treatment only after the mandate. There were 400 cycles of TCHL treatment given to patients on the trial prior to the loperamide mandate resulting in 109 diarrhoea-associated events. However, after the loperamide mandate, 200 cycles of TCHL treatment were given resulting in 36 events. The average rate before the loperamide mandate was approximately 0.72 events per cycle compared to an average rate of 0.27 events per cycle after the mandate. A comparison between these two rates resulted in a significant relative reduction of 64% in the rate of diarrheal events after the mandate (95% CI: 18.4–83.8), in patients who received TCHL/TCL (*p* = 0.007).

### Translational sub-study

Using unique immunohistochemistry (IHC) data generated previously [20], tumour CD3+, CD4+, and CD8+ immune cell counts at baseline (diagnostic biopsy) and pre-cycle 2 (on-treatment biopsy) were investigated in the context of three key measures of peripheral oxygen carrying capacity taken pre-cycle 1, pre-cycle 2, and at the end of six cycles of therapy (End chemo/pre-surgery). Results show that baseline tumour CD3+ T-cell levels do not correlate with blood counts at any timepoint. However, on-treatment (pre-cycle 2), tumour CD3+ T cell levels positively correlate with Pre-cycle 2 and End Chemo RBC, haemoglobin, and haematocrit counts (Table 5). Tumour CD4+ T-cell results were consistent with these outcomes for haemoglobin and haematocrit Pre-Cycle 2 and End chemo (Supplementary Table 1). Tumour CD8+ T cells were significantly correlated with RBC, haemoglobin, and haematocrit counts for the End chemo timepoint only (Supplementary Table 2). Results suggest that tumour T-cell count may be associated with circulating oxygen levels during therapeutic intervention, but not in advance of this intervention.

**Table 5:** Correlative analysis of tumour CD3+ immune cells (Baseline, pre-cycle 2 by immunohistochemistry) with red blood cell (RBC) count, haemoglobin and haematocrit levels (Pre-cycle 1, Pre-cycle 2 and End chemo) in 13 patients (*n* = 8 pCR, *n* = 5 No pCR) from the TCHL study.

pCR response		Tumour CD3+ cell count baseline		Tumour CD3+ cell count pre-cycle 2	
		*P*	Adjusted *P*	*P*	Adjusted *P*
Pre-cycle 1	RBC	0.783	0.881	0.543	0.698
	Haemoglobin	0.728	0.881	0.820	0.852
	Haematocrit	0.766	0.881	0.852	0.852
Pre-cycle 2	RBC	0.163	0.850	0.032*	0.048*
	Haemoglobin	0.313	0.850	0.015*	0.032*
	Haematocrit	0.227	0.850	0.031*	0.048*
End chemo	RBC	0.921	0.921	0.005*	0.033*
	Haemoglobin	0.378	0.850	0.011*	0.033*
	Haematocrit	0.514	0.881	0.008*	0.033*

Spearman rank-order correlation coefficient, *p* values adjusted using Bonferroni correction.

* *P* < 0.05, statistically significant. All significant values were positively correlated. pCR: pathological complete response.

## Discussion

The study did not meet its primary endpoint, that is the addition of L to neoadjuvant chemotherapy and H did not increase the proportion of patients who achieved pCR.

While our H and L pCR rate of 51.6% is comparable to that of other similar studies, which ranged from 34.8% in GeparSixto [22] to 62% in NSABP-B41 [23], we report a higher-than-expected frequency of pCR in our control (i.e. H only) Arm A. Our Arm A (H-alone) pCR rate at 52.8% was numerically higher than that observed in all other studies which ranged from 25% in CherLob [6] to 52.5% in NSABP-B41 [23].

The overall pCR rate achieved across both arms of our trial was comparable to other neoadjuvant studies which included H and/or L. In the NeoALTTO [5], TRIO-B07 [24], Cherlob [6], NSABP-B41 [23], CALGB40601 [25], GeparQuinto [26], and GeparSexto [22] the overall pCR rates ranged from 32% in CherLob [6] to 55.9% in NSABP B41 [23].

Some but not all published trials have demonstrated clinical benefits for dual targeting of HER2 with H and L in the neo-adjuvant setting compared to H alone [5, 6, 22–26]. A recent meta-analysis (which did not include our study) demonstrated a positive impact on both DFS and OS [27]. Pre-clinical work has provided *in vitro* evidence for both signalling and immune-associated mechanisms for the basis of H and L synergy [17, 28, 29].

Differences in overall pCR rates do not appear to be attributable to the different chemotherapy arms tested. For example, the NSABP-B41 study that had the highest pCR rate (55.9%) included a chemotherapy backbone of doxorubicin, cyclophosphamide, and paclitaxel [23], whilst the GeparQuinto study (pCR rate of 27%) included docetaxel, epirubicin, and cyclophosphamide [26]. Studies that included both docetaxel and carboplatin (TRIO B-07 and TCHL) had comparable pCR rates overall (47% vs 44%) [24].

We have previously demonstrated the importance of timing of H delivery as a determinant of survival [30]. However in the NSABP B41 study (which had the highest pCR rates respectively) chemotherapy was given to patients prior to them receiving H.

Patients in our trial who were treated with H and L, had a significantly superior 5-year DFS compared to those patients who received H alone. In a meta-analysis conducted by Guarneri et al. (2022), it was reported that when individual studies that included HL treated patients were analysed (NeoALTTO, NSABP-41 and CHERLOB) only those patients in the CHERLOB study had significantly improved OS [27]. However the meta-analysis of these three studies showed that HL had a significantly higher OS rate when compared to H alone [27]. In relation to our study, longer follow-up may be needed to observe significant improvements in OS.

During enrolment to the TCHL trial, when evidence emerged of a high incidence of diarrhoea events in patients who receive L [31, 32], we mandated that loperamide should be included in the in the L arms. Loperamide significantly reduced the incidence and severity of diarrhoea across all treatment arms.

A link between the anti-tumour immune response and response to therapy has been a consistent theme in translational studies from the TCHL trial. Translational studies on circulating immune cells from TCHL study patients identified that PD-1-inhibited ADCC-capable immune cells were associated with lower baseline TIL levels as a potential biomarker of response to therapy, providing evidence that immune suppression has a role to play in pCR to neo-adjuvant therapy in HER2+ breast cancer [33]. Analysis of TILs within TCHL tumour samples was published in 2021 [20]. The study reported that patients who achieved a pCR had higher levels of TILs than those who did not. A novel aspect of the TIL study was the examination of CD3+, CD4+, and CD8+ immune cell subpopulations in pre-treatment diagnostic, and on-treatment (pre-cycle 2) biopsies in a subset of patient samples. This analysis revealed that decreased numbers of CD4+ and CD3+ T-cells correlated with a reduction in tumour volume [20], again emphasising the role of the immune response in this setting.

Myelosuppressive chemotherapies, including docetaxel and cisplatin, are known to cause anaemia [34]. Hypoxia in the tumour microenvironment has been shown to reduce T-cell activity, leading to therapy resistance and tumour escape [35]. The preliminary data presented in this manuscript suggests an association between tumour-associated T cells and RBC/haemoglobin levels, but only after treatment is initiated. Tissue oxygen is a key regulator of the immune response, directly affecting immune effector energy status and the intensity of regulatory activity [36]. Peripheral haemoglobin levels have been previously associated with treatment outcome, but not tumour T cell levels, in breast cancer [37–39]. Haemoglobin levels have also been reported as a prognostic factor in other cancer types such as clear cell renal cancer [40] and small cell lung cancer [41]. Hypoxia in the tumour microenvironment leads to the promotion of angiogenesis, metastasis, tumour cell stemness, resistance to radio/chemotherapy, and immune suppression [35]. While high RBC, haematocrit, and haemoglobin counts are an indication of general patient well-being, it may also hint that optimising peripheral oxygen capacity during therapy may have therapeutic application. We include the translational study here to highlight an interesting, preliminary insight into a previously unreported observation linking peripheral oxygen carrying capacity with TIL levels following the commencement of treatment. While the numbers are limited in this analysis and should not be over-interpreted, testing the observation in larger datasets with varying chemotherapy backbones and with routine patient blood and TIL data available, would provide the opportunity to further explore the finding [33].

Whilst L is not the standard of care for early stage HER2+ breast cancer patients [42], recent studies demonstrate that dual inhibition of HER2 using H and L results in improved DFS and OS [27]. It has been long known that the combination of TCH and pertuzumab (TCHP) improves both pCR rates in the neoadjuvant setting [42].

However the analysis of cost effectiveness of the combination of TCHP has mixed reviews. In one study from Canada it was deemed cost-effective in all patients [43]; however, in the USA another study determined it was only cost effective in high-risk patients [44], whilst it was deemed not cost-effective in lower/middle income countries such as Columbia [45].

In summary our study, which did not meet its primary endpoint of improved pathological complete remission with L plus H versus H alone, did show a statistically significant improvement in DFS for HL compared to H, but without an OS advantage. The use of loperamide significantly reduced the frequency of diarrhoea in the combination. Our preliminary translational study findings suggest that peripheral oxygen capacity should be investigated further in the context of tumour immune cell infiltrate during therapy. Whilst the TCHL trial has a relatively small number of participants, its data strongly correlate with recently published clinical data, and suggest that anti-HER2 TKIs merit further investigation in the neo-adjuvant treatment of low-risk early stage breast cancer patients.

## Supplementary Material

TCHL – a phase II neo-adjuvant study assessing TCH (docetaxel, carboplatin and trastuzumab) and TCHL (docetaxel, carboplatin, rastuzumab and lapatinib) in HER-2 positive breast cancer patients: a 5-year follow-up with serum biomarker analysis

## Data Availability

The datasets generated and/or analysed during the current study are available from the corresponding author with approval from the study sponsor on reasonable request.
