# Supplementary material for: TCHL – a phase II neo-adjuvant study assessing TCH (docetaxel, carboplatin and trastuzumab) and TCHL (docetaxel, carboplatin, rastuzumab and lapatinib) in HER-2 positive breast cancer patients: a 5-year follow-up with serum biomarker analysis
Source: Acta Oncol. 2025 Jun 5;64:43143. doi: 10.2340/1651-226X.2025.43143 (PMC12171739; doi:10.2340/1651-226X.2025.43143)
Supplement: TCHL – a phase II neo-adjuvant study assessing TCH (docetaxel, carboplatin and trastuzumab) and TCHL (docetaxel, carboplatin, rastuzumab and lapatinib) in HER-2 positive breast cancer patients: a 5-year follow-up with serum biomarker analysis [file AO-64-43143-s1.pdf]

Supplementary material has been published as submitted. It has not been copyedited, or typeset by Acta Oncologica

A

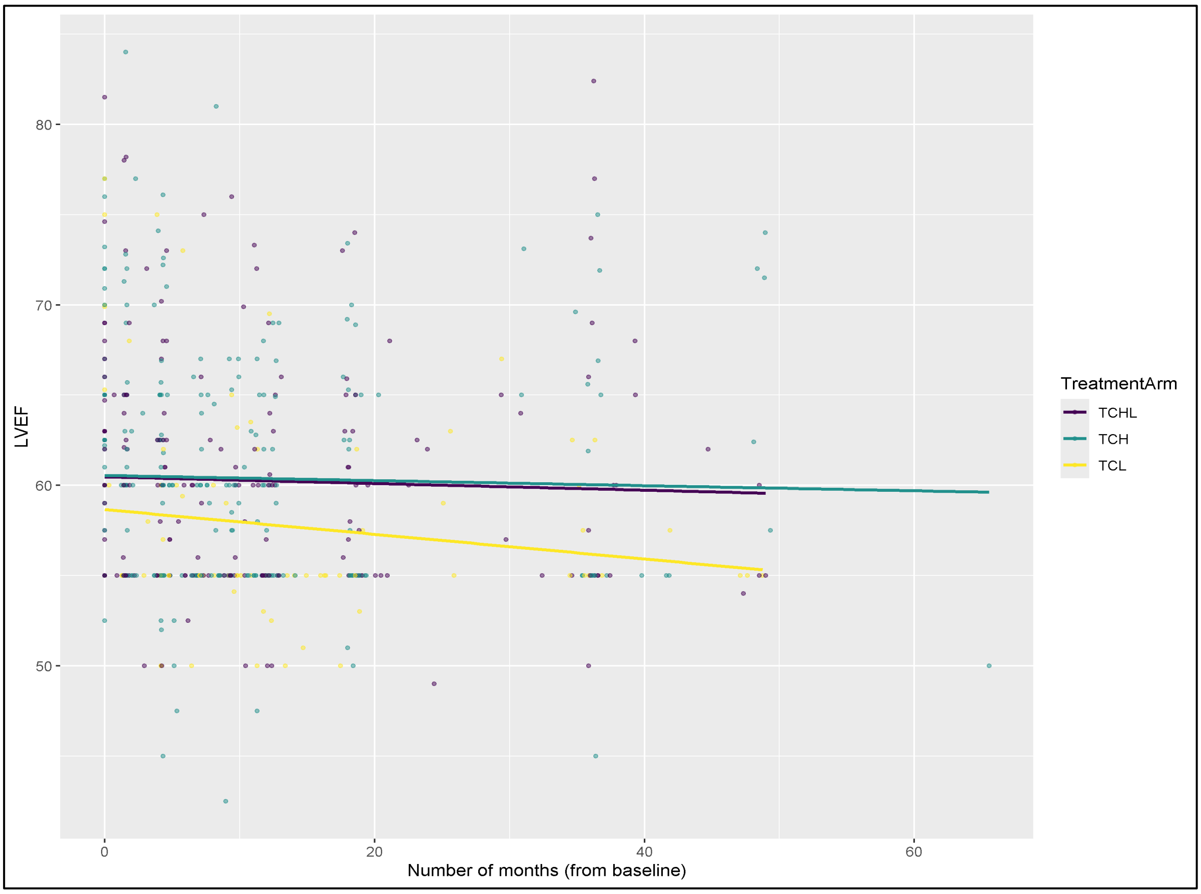

B

TCH Patient	PT	SOC	AE Grade	Serious	Outcome
**1**	Cardiac disorder	Cardiac disorders	1	No	Resolved
**2**	Cardio-respiratory arrest; Tachycardia; Cyanosis	Cardiac disorders	5	Yes	Fatal
**3**	Palpitations	Cardiac disorders	1	No	Resolved
**4**	Acute myocardial infarction	Cardiac disorders	3	Yes	Resolved

C

TCHL Patient	PT	SOC	AE Grade	Serious	Outcome
**1**	Palpitations	Cardiac disorders	1	No	Resolved
**2**	Palpitations	Cardiac disorders	1	No	Resolved
**3**	Palpitations	Cardiac disorders	2	No	Resolved

Supplementary Figure 1: A) Analysis of left ventricular ejection fraction (LVEF) over the course of the TCHL clinical study as per treatment arm. B) Adverse Cardiac events recorded for TCH treated patients. C) Adverse Cardiac events recorded for TCHL treated patients. No TCL treated patients had any recorded cardiac adverse events.

Supplementary Table 1. Correlative analysis of tumour CD4+ immune cells (Baseline, pre-cycle 2 by immunohistochemistry) with red blood cell (RBC) count, haemoglobin and haematocrit levels (Pre-cycle 1, Pre-cycle 2 and End chemo) in 13 patients (n=8 pCR, n=5 No pCR) from the TCHL study. Spearman rank-order correlation coefficient, p values adjusted using Bonferroni correction. * $p < 0.05$, statistically significant. All significant values were positively correlated.

		Tumour CD4+ cell count Baseline		Tumour CD4+ cell count Pre-cycle 2	
		p value	Adjusted p value	p value	Adjusted p value
Pre-cycle 1	RBC	0.499	0.837	0.869	0.869
	Haemoglobin	0.322	0.837	0.608	0.715
	Haematocrit	0.266	0.837	0.635	0.715
Pre-cycle 2	RBC	0.482	0.837	0.115	0.207
	Haemoglobin	0.706	0.837	0.007 *	0.027 *
	Haematocrit	0.774	0.837	0.005 *	0.027 *
End chemo	RBC	0.499	0.837	0.201	0.302
	Haemoglobin	0.837	0.837	0.009*	0.027*
	Haematocrit	0.604	0.837	0.030*	0.068

Supplementary Table 2. Correlative analysis of tumour CD8+ immune cells (Baseline, pre-cycle 2 by immunohistochemistry) with red blood cell (RBC) count, haemoglobin and haematocrit levels (Pre-cycle 1, Pre-cycle 2 and End chemo) in 13 patients (n=8 pCR, n=5 No pCR) from the TCHL study. Spearman rank-order correlation coefficient, p values adjusted using Bonferroni correction. * $p < 0.05$, statistically significant. All significant values were positively correlated.

		Tumour CD8+ cell count Baseline		Tumour CD8+ cell count Pre-cycle 2	
		p value	Adjusted p value	p value	Adjusted p value
Pre-cycle 1	RBC	0.237	0.559	0.366	0.366
	Haemoglobin	0.278	0.559	0.268	0.344
	Haematocrit	0.287	0.559	0.308	0.347
Pre-cycle 2	RBC	0.373	0.559	0.129	0.195
	Haemoglobin	0.679	0.764	0.029*	0.066
	Haematocrit	0.350	0.559	0.057	0.103
End Chemo	RBC	0.331	0.559	0.003*	0.014*
	Haemoglobin	0.991	0.991	0.005*	0.014*
	Haematocrit	0.604	0.764	0.001*	0.009*
